# Benchmarking Large Language Models Using a Best Evidence Topic Report in a Patient With Early Non-Small Cell Lung Cancer

**DOI:** 10.1093/icvts/ivag038

**Published:** 2026-02-06

**Authors:** Vivek Chaudhuri, Alessandro Brunelli, Peter Tcherveniakov, Nilanjan Chaudhuri

**Affiliations:** University of Oxford, Oxford, OX2 6QA, United Kingdom; University of Leeds, Leeds, LS2 9JT, United Kingdom; Thoracic Surgery, St. James University Hospital, Leeds, Bexley Wing Level 3, LS9 7TF, United Kingdom; University of Leeds, Leeds, LS2 9JT, United Kingdom; Thoracic Surgery, St. James University Hospital, Leeds, Bexley Wing Level 3, LS9 7TF, United Kingdom; University of Leeds, Leeds, LS2 9JT, United Kingdom; Thoracic Surgery, St. James University Hospital, Leeds, Bexley Wing Level 3, LS9 7TF, United Kingdom

**Keywords:** RATS—robotic-assisted thoracoscopic surgery, VATS—video-assisted thoracoscopic surgery, NSCLC—non-small cell lung cancer, ChatGPT, Gemini, Grok, Microsoft Copilot

## Abstract

**Objectives:**

Large language models (LLMs) are generative-AI which generate text output like a human conversation. We wanted to assess the ability of LLMs to answer patient’s questions and benchmark their output using a best evidence topic (BET).

**Methods:**

We asked LLMs whether robot-assisted thoracic surgery (RATS) or video-assisted thoracoscopic surgery (VATS) lobectomy had better perioperative outcomes for postoperative pain, length of hospital stay (LOS) and mortality. A BET was constructed according to a structured protocol for the same questions. An initial search yielded 324 papers, 12 represented the best evidence.

**Results:**

LLM outputs are almost instantaneous while a BET took many hours of searching a database for relevant evidence. However, current iterations and models of LLMs did not provide relevant outputs, suffered from hallucinations, and could be restricted by copyright and paywall issues. The BET, on the other hand, was tailored to the scenario by specialist human oversight and therefore more reliable and nuanced.

**Conclusions:**

There were no major differences between RATS and VATS lobectomy for T1cN0M0 NSCLC apart from shorter LOS following RATS. Current LLMs may not be entirely reliable for answering clinical questions. An LLM-BET protocol could be used as a standardized process to compare LLM outputs for different clinical scenarios, each benchmarked with a BET. It can also be used to analyse outputs of different models of current and future LLMs.

## Graphical Abstract

**Figure:**
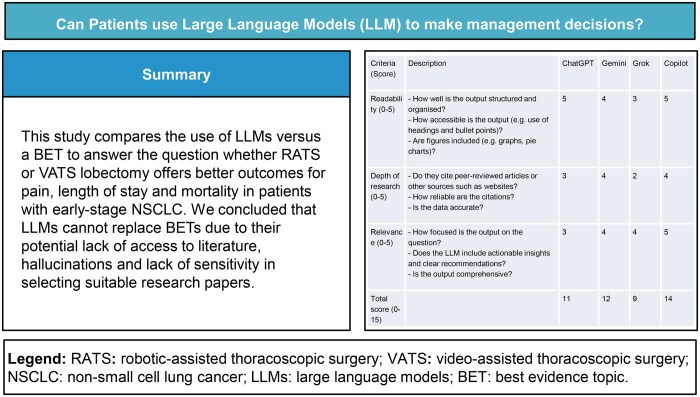

## INTRODUCTION

Large language models (LLMs) are a subgenre of generative artificial intelligence (Gen AI), which produce textual output with a contextual and conversational format.^1^^,^^2^ They have the potential to aid clinical decision-making.^2^^,^^3^ LLMs have also become popular amongst the general public^4^ and might help them better understand a disease and its management. We wanted to assess the reliability and accuracy of LLMs as tools for patients asking relevant clinical questions.

We created a plausible clinical patient scenario for a patient with early lung cancer. Chat GPT, Gemini, and Microsoft Copilot are popular LLMs in 2025.^5–7^ Grok is an LLM integrated with the social microblogging service X (formerly Twitter).^8^ We asked these LLMs a series of questions related to the scenario and compared their output using pre-agreed metrics (Table 1). In addition to a general comparison between robot-assisted thoracic surgery (RATS) and video-assisted thoracoscopic surgery (VATS) lobectomy, we decided to focus on 3 specific outcomes (pain, length of stay, and mortality) that a patient might be interested in.

**Table 1.** LLM Output Assessment Metrics

Criteria (score)	Description	ChatGPT	Gemini	Grok	Copilot
Readability (0-5)	How well is the output structured and organized? How accessible is the output (eg, use of headings and bullet points)? Are figures included (eg, graphs, pie charts)?	5	4	3	5
Depth of research (0-5)	Do they cite peer-reviewed articles or other sources such as websites? How reliable are the citations? Is the data accurate?	3	4	2	4
Relevance (0-5)	How focused is the output on the question? Does the LLM include actionable insights and clear recommendations? Is the output comprehensive?	3	4	4	5
Total score (0-15)		11	12	9	14

We also constructed a best evidence topic (BET) based on the clinical scenario and the chosen outcomes, according to an agreed protocol.^9^ We compared LLM outputs to a BET, which is designed to answer a specific clinical question. Asking an LLM the same question tests its ability to selectively identify relevant sources of information. Additionally, this comparison should inform clinicians about the capabilities of LLMs to influence patient decisions. A standardized LLM-BET protocol can also be used to both improve and critique advancements of iterations of LLMs in future.

## CLINICAL SCENARIO

A 63-year-old retired paramedic is informed that he has an early lung cancer in the right upper lobe of his lung. It is staged as T1cN0M0 non-small cell lung cancer (NSCLC), and the patient is offered VATS for resection of the tumour. However, the paramedic is aware that an alternative modality of surgery using a surgical robot (RATS) is also available, so they do a search using an LLM to see which surgical approach is recommended. At the next appointment, the paramedic asks the clinician whether robotic lung resection is an option. The clinician then decides to assess the available evidence in the form of a BET and compare it with the output of LLMs before further discussion with the patient.

## LARGE LANGUAGE MODELS

A set of 5 questions that a patient with limited clinical awareness of their condition might ask these generative AI models was created.

1. Is robotic or VATS lobectomy better for a T1cN0M0 lung cancer?
2. Am I likely to stay in in the hospital longer after a robotic lobectomy or VATS lobectomy for lung cancer?
3. Am I less likely to die after a robotic or VATS lobectomy for T1cN0M0 lung cancer?
4. Am I more likely to die in the hospital or in the first thirty days after a robotic or a VATS lobectomy for a T1cN0M0 lung cancer?
5. Am I likely to experience more postoperative pain after a robotic or VATS lobectomy for a T1cN0M0 lung cancer?

We then posed the same questions to 4 LLMs (ChatGPT, Gemini, Grok, and Microsoft Copilot—**Appendix S1**) without any prior context injection. There was some variability between the LLMs, as some offered to produce additional outputs. If these were relevant to our study, we answered yes. As an example, Copilot offered to draft a series of helpful questions for the patient to ask their thoracic surgeon, and ChatGPT offered to show the 5-year survival curves for RATS vs VATS from a meta-analysis. We then assessed the LLM responses according to pre-agreed metrics (**Table 1**). Three metrics (Readability, Depth of research, and Relevance) made the final shortlist, and each metric was scored between 0 and 5 based on clearly described (and agreed upon) criteria (see **Table 1**). Therefore, the maximum score any LLM could achieve was 15.

ChatGPT was awarded an overall score of 11. Its readability scored 5 (maximum allowed) due to the clean layout, incorporation of bullet points, and bold formatting where relevant. It also used tables and a graph to present data in a visually appealing and easily understandable manner. For depth of research, ChatGPT mainly cited peer-reviewed articles (**Appendix S2**), with the majority focusing on lobectomy. However, some of the articles included data from sublobar resections, and the majority did not focus solely on stage I NSCLC patients. Upon a closer look at the data, it became clear that ChatGPT was sometimes incorrect as it made up values. These are recognized issues with LLMs, called hallucinations.^10–12^ Considering all of these factors, we decided to give ChatGPT a score of 3 for depth of research. ChatGPT remained focused on the input question but did not offer any actionable insights, such as talking to your thoracic surgeon, giving it a score of 3 for relevance.

Gemini scored 12. Gemini would have scored 3 for readability as its initial report did not highlight keywords and important phrases and lacked other visual aids such as tables or graphs. However, it did offer to produce a research report and executive summary for Question 1 (general comparison between RATS and VATS lobectomy). This latter report (**Appendix S1**; Page 20) was excellent and included a table, and therefore, we upgraded the score to 4. Gemini scored 4 for depth of research as it referenced some peer-reviewed articles with accurately referenced data. However, some included sublobar resections, and the majority did not focus solely on stage I NSCLC patients. For relevance, Gemini scored 4 as it remained focused on the input question and included a discussion of the decision-making process and how it is based on collaboration between the surgeon and patient.

Grok’s overall score was 9. Grok scored a 3 for readability due to a lack of highlighted keywords and phrases and summary tables. Despite citing somewhat relevant RCTs such as the RVLOB,^13^ BRAVO,^14^ and PORTaL^15^ studies, Grok scored a 2 for depth of research due to incorrect citations of literature (perhaps hallucinations) linking to irrelevant studies^16–19^ which could mislead the lay reader. Besides, just like ChatGPT and Gemini, Grok also included articles discussing sublobar resection and articles which didn’t solely focus on stage I NSCLC patients. For relevance, we gave Grok a score of 4 as it remained focused on the input question and offered helpful recommendations, such as the importance of discussing comorbidities with your thoracic surgeon.

Copilot scored 14 overall. For readability, Copilot scored 5 due to a helpful inclusion of tables, highlighted keywords, and short, succinct bullet-pointed phrases. Although Copilot mainly cited peer-reviewed articles and correctly referenced data, it did include articles discussing sublobar resection and articles which didn’t solely focus on stage I NSCLC patients, just like all the above LLMs. Therefore, we gave it a score of 4 for depth of research. Copilot scored 5 for relevance as it remained focused on the input question. It also offered to draft a series of helpful questions for the patient to ask their thoracic surgeon to aid the decision-making process (**Appendix S3**).

## BET—THREE-PART QUESTION

Our 3-part question for the chosen clinical scenario was “In patients with [early lung cancer] does [VATS or RATS lobectomy] offer [better outcomes for pain, length of stay and mortality]?”

## SEARCH STRATEGY

Medline 1946-Sep 2025 was searched using the Ovid interface. [(robot* or robot-assisted thoracic surgery or robot-assisted thoracoscopic surgery or RATS).mp.] AND [VATS.mp. or exp Thoracic Surgery, Video-Assisted/or video-assisted thoracoscopic surgery.mp. or video-assisted thoracic surgery.mp.] AND [(outcome* or length of stay or hospital stay or pain or mortalit*).mp. or exp mortality/or exp outcome/] AND [NSCLC.mp. or exp Carcinoma, Non-Small-Cell Lung/or non-small-cell lung.mp. or early.mp. or stage I*.mp.].

## SEARCH OUTCOME

A total of 324 papers were identified using the above search. Papers not specifically reporting on stage I lung cancer, lobectomy, or the 3 chosen outcomes were excluded, along with review papers and non-English papers. The remaining 12 papers represented the best evidence for this topic and are presented in **Table 2**.

**Table 2.** Best Evidence Papers

Author, date, journal, study type	Paper title	Patient group	Outcomes	Key results	Comments
Zhang et al., 2025, *Clin Lung Canc*,^20^ Retrospective study	Comparison of long-term survival between robotic and video-assisted lobectomy for Stage I NSCLC with radiologic solid tumors: A propensity score matching study.	Single-institution retrospective study of 518 patients with c-stage I NSCLC undergoing lobectomy (225 RATS, 293 VATS). After propensity score matching (PSM), 170 pairs of cases were matched between the RATS and VATS groups.	Postoperative hospital length of stay (LOS), 5-year overall survival (OS).	Mean postoperative LOS (days) was 5.31 for RATS and 5.60 for VATS, *P* = .308. 5-year OS rate was 92% for RATS and 89% for VATS, *P* = .620	Perioperative mortality was not mentioned, but at 0 months (Kaplan-Meier), RATS 162 patients (8 deaths) and VATS 163 patients (7 deaths).
Casiraghi et al., 2022, *J Clin Med*,^21^ Retrospective study	Long-term outcomes of robotic-assisted, video-assisted and open surgery in non-small cell lung cancer: A matched analysis.	Single-institution retrospective study of 561 consecutive patients with stage I NSCLC who underwent lobectomy (49 VATS, 254 RATS, 258 open). After PSM, there were 180 patients (72 RATS, 36 VATS, 72 open).	LOS, 5-year OS, 90-day mortality.	Median LOS (days) was 5 for RATS vs 6 for VATS and 6 for open (*P* < .05). 5-year OS, % (95% CI) was 77.3 (59.7-88.0) for VATS vs 87.4 (75.8-93.7) for RATS and 78.6 (67.0-86.5) for open, with no statistically significant difference.	There were no deaths within 90 days of operation in either the VATS, RATS or Open group.
Yang et al., 2017, *Ann Surg*,^22^ Retrospective study	Long-term survival based on the surgical approach to lobectomy for clinical Stage I non-small cell lung cancer: comparison of robotic, video-assisted thoracic surgery, and thoracotomy lobectomy.	Single-institution retrospective study of 2132 patients with clinical stage I NSCLC who underwent a lobectomy (184 RATS, 761 VATS, 1187 open). After PSM, there were 516 cases included (172 RATS, 172 VATS, 172 open).	LOS, 5-year OS.	LOS, days, median (range) was 4 (1-32) for RATS, 4 (2-50) for VATS and 5 (2-29) for open, *P* < .001 5-year OS was 77.6% for RATS, 73.5% for VATS, 77.9% for open with no significant difference in OS.	80% of patients in unmatched RATS and VATS groups were Stage 1 A. In PSM groups no death after RATS and 1 (operative) death after VATS in the first 90 days.
Cui et al., 2020, *JNCI Cancer Spectr*,^23^ Retrospective study	Mortality for robotic- vs video-assisted lobectomy-treated Stage I non-small cell lung cancer patients.	Retrospective study of 18 908 patients from the National Cancer Database (NCDB) with stage I NSCLC undergoing a lobectomy (14279 VATS, 4629 RATS).	Long-term total mortality.	90-Day mortality (with or without conversion) was 288 patients (2.0%) for VATS vs 110 (2.4%) for RATS, *P* = .14. If conversion to open thoracotomy occurred, 90-day mortality was 6.3% for RATS vs 3.8% for VATS, *P* = .03. There was an increased long-term all-cause mortality risk for RATS vs VATS when tumour size was ≤20 mm, *P* interaction = 0.007 to 0.02 (statistically significant interaction between RATS and VATS and tumour size on long-term mortality risk).	Cases were selected between 2010 and 2014 (early RATS experience). RATS constituted only a fourth of patients.
Li et al., 2019, *J Thorac Dis*,^24^ Retrospective study	Perioperative outcomes of radical lobectomies using robotic-assisted thoracoscopic technique vs video-assisted thoracoscopic technique: retrospective study of 1,075 consecutive p-stage I non-small cell lung cancer cases.	Retrospective study of 1075 patients with stage I NSCLC who underwent a lobectomy (237 RATS, 838 VATS) by the same surgical team. After PSM, 230 pairs of patients were matched between RATS and VATS.	LOS, 30-day mortality.	Mean LOS (SD) for RATS was 4.97 (1.56) vs 5.45 (2.01) for VATS, *P* .004.	30-Day mortality: In unmatched group no death after RATS, not specified after VATS (as grouped with readmission). After PSM no 30 day deaths in either RATS or VATS patients.
Testori et al., 2022, *J Clin Med*,^25^ Retrospective study	Robotic and video-assisted thoracic surgery for early-stage lung cancer: comparison of long-term pain at a single centre.	Single-centre study of 100 patients (50 RATS, 50 VATS) who underwent lobectomies for clinical stage I NSCLC.	Postoperative pain.	Pain recorded using a Numerical Rating Scale (NRS). Median NRS scores were similar between RATS and VATS groups 2 weeks, 3 months, 6 months and 1 year after surgery. 2 weeks: NRS value for RATS was 2.86 (SD = 1.05) vs 2.96 (SD = 0.83) for VATS. 3 months: NRS value for RATS was 2.06 (SD = 0.87) vs 2.16 (SD = 0.65) for VATS. 6 months: NRS value for RATS was 1.56 (SD = 0.67) vs 1.62 (SD = 0.64) for VATS. 1 year: NRS value for RATS was 1.24 (SD = 0.47) vs 1.30 (SD = 0.54) for VATS. No statistically significant difference between pain scores at each time interval (*P* > .05)	
Hennon et al., 2020, *Eur J Cardiothorac Surg*,^26^ Retrospective study	The association of nodal upstaging with surgical approach and its impact on long-term survival after resection of non-small cell lung cancer.	Retrospective study of 46 826 patients from the NCDB who underwent lobectomy (4338 RATS, 13416 VATS, 29072 open) for clinical stage I NSCLC.	OS, 30/90 day mortality and LOS	No significant difference in OS between RATS and VATS, log-rank *P* = .0651. 30/90-day mortality (%) was 1.6/2.6 for RATS, 1.4/2.3 for VATS. For 30-day mortality, *P* = 0.432 and for 90-day mortality, *P* = .530. 5-Year survival (hazard ratio) was 0.67 (0.63-0.71) for RATS, 0.72 (0.71-0.74) for VATS, 0.67 (0.66-0.68) for open, *P* < .01. LOS, median (days) was 4 for RATS, 5 for VATS, 6 for open, *P* < .01.	Patients operated between 2010 and 2014 30/90-day mortality was lower for minimally invasive (RATS and VATS) groups than for open patients in both unmatched and after PSM.
Yang et al., 2016, *Ann Thorac Surg*,^27^ Retrospective study	Use and outcomes of minimally invasive lobectomy for Stage I non-small cell lung cancer in the National Cancer Data Base.	Retrospective study of 30040 patients from the NCDB with stage I (clinical T1-2, N0, M0) NSCLC who underwent a lobectomy (7824 VATS, 2025 RATS, 20191 open). After PSM, 1938 pairs of patients were matched between RATS and VATS.	LOS, 30-day mortality, 2-year OS.	LOS, days, was 5 (3-7) for VATS vs 5 (3-7) for RATS, *P* = .34. 30-day mortality was 17 (1.5) for VATS vs 12 (1.3) for RATS, *P* = .96. 2-year OS % (95% CI) was 86% (84% to 88%) for VATS vs 85.3% (83% to 88%) for RATS, *P* = .9.	
Niu et al., 2024, *EClinicalMedicine*,^13^ Randomized controlled trial	Robotic-assisted versus video-assisted lobectomy for resectable non-small-cell lung cancer: the RVlob randomized controlled trial.	Randomized controlled trial of 320 patients with c-stage IA-IIIA NSCLC, randomized to undergo RATS or VATS lobectomy (157 RATS, 163 VATS).	OS.	After post-hoc subgroup analysis on pTNM stage I NSCLC patients, hazard ratio (95% CI) was 0.86 (0.35 to 2.11), *P* = 0.737 for RATS vs VATS (hazard ratio < 1 implies lower risk of overall survival after RATS lobectomy vs VATS lobectomy).	No mention of perioperative mortality. 1 year OS for all patients: 100% in RATS and 96.8% in VATS group (supplemental Table 2 in [Niu 2024]). Stage I patients 82.8%, (265/320). Subgroup analysis was performed on pTNM stage I for overall survival.
Jin et al., 2023, *Chest*,^28^ Randomized control trial	Health-related quality of life following robotic-assisted or video-assisted lobectomy in patients with non-small cell lung cancer.	Randomized controlled trial of 320 patients with c-stage IA-IIIA NSCLC, randomized to undergo RATS or VATS lobectomy (157 RATS, 163 VATS).	Postoperative pain.	VAS (visual analogue scale) was used for assessing pain on postoperative day 1 and NRS (numerical rating scale) was used for baseline evaluation of pain and at weeks 4, 24 and 48. Patients who had undergone VATS lobectomy reported significantly higher pain scores compared to RATS lobectomy at week 4. Subgroup analysis produced a *P*-value of .05 for pTNM stage I NSCLC patients at week 4 (difference in pain between RATS and VATS at week 4 is not statistically significant).	RVlob trial. Subgroup analysis performed on pTNM stage I for postoperative pain.
Fabbri et al., 2023, *J Clin Med*,^29^ Prospective study	Long-term oncologic outcomes in robot-assisted and video-assisted lobectomies for non-small cell lung cancer.	Single-centre prospective study of 619 patients with c-stage I-III NSCLC who underwent lobectomy (403 RATS, 216 VATS)	OS.	After subgroup analysis on pTNM stage I NSCLC patients: 3-year OS was 86.8% for VATS and 86.3% for RATS. 5-year OS was 75.7% for VATS and 83.4% for RATS *P* = 0.436.	Paper included as Subgroup analysis performed on pTNM stage I for overall survival. No subgroup analysis performed on 30-day mortality for Stage I patients. Overall, no significant difference between RATS and VATS
Kneuertz et al., 2020, *Clin Lung Cancer*,^30^ Retrospective study	Long-term oncologic outcomes after robotic lobectomy for early-stage non-small-cell lung cancer versus video-assisted thoracoscopic and open thoracotomy approach.	Single-centre retrospective study of 540 patients with stage I-III NSCLC undergoing lobectomy. After PSM, 514 patients were included (245 RATS, 118 VATS, 151 open).	OS.	For stage 0/Ia patients, 3-year survival (95% CI) was 0.86 (0.76-0.92) for RATS, 0.76 (0.57-0.87) for VATS, 0.80 (0.65-0.89) for open. Overall *P* value = .13. For stage Ib patients, 3-year survival (95% CI) was 0.82 (0.71-0.90) for RATS, 0.85 (0.66-0.93) for VATS, 0.75 (0.56-0.87) for open, *P* = .88. For stage Ib patients, 5-year survival (95% CI) was 0.67 (0.52-0.79) for RATS, 0.70 (0.35-0.89) for VATS, 0.65 (0.42-0.80) for open. For stage Ib patients, overall *P* value = 0.88.	5-year survival for patients with stage 0/Ia disease who underwent VATS was not estimated in the study.

Abbreviations: CI, confidence interval; LOS, length of stay; OS, overall survival; PSM, propensity score matching.

## RESULTS

### Mortality and survival

The overwhelming majority of the 9 BET studies discussing mortality and survival showed no significant difference between RATS and VATS (Table 2). Cui *et al.*^23^ determined that RATS has an increased long-term mortality risk only for tumour sizes ≤20 mm (T1a or T1b). Tumours >20 mm did not show any significant difference. Hennon *et al.*^26^ produced a statistically significant result. However, as this study included a comparison to open thoracotomy we cannot reach any definite conclusions about the difference in mortality between RATS and VATS.

ChatGPT stated that the risk of death after RATS or VATS is extremely low, suggesting that more important factors affecting survival are the stage and biology of the tumour, quality of surgical resection, and the patient’s baseline health and lung function. For 30-day mortality, ChatGPT concluded that there is no demonstrable difference between RATS and VATS, although it incorrectly referenced data from 2 meta-analyses.^31^^,^^32^ ChatGPT offered to show the percentage mortality by stage. Correctly referencing data,^23^ it stated that for stage I NSCLC, after conversion to thoracotomy, RATS has a significantly higher 90-day mortality compared to VATS.

Gemini stated there is no significant difference in long-term overall survival between RATS and VATS, citing one randomized-controlled trial^33^ and another study.^34^ However, it did acknowledge conflicting data from the PORTaL study,^35^ which found a better overall survival for RATS compared to VATS and a meta-analysis which found a lower 30-day mortality^36^ for RATS.

Grok concluded that RATS and VATS have comparable mortality rates, citing the RVlob trial^13^ but incorrectly referencing its data. According to Grok, long-term mortality is more influenced by the tumour biology and adjuvant therapy, as complete resection is achieved in > 95% of cases with RATS or VATS. Grok did not offer any citation to support this claim.

Copilot cited the same article as ChatGPT,^23^ stating that RATS has a higher 90-day mortality compared to VATS if the operation was converted to open thoracotomy. Copilot also cited a meta-analysis^32^ suggesting there is no significant difference in overall survival between RATS and VATS. For 30-day mortality, Copilot cited a meta-analysis^37^ which suggested there is a significantly lower 30-day mortality for RATS compared to VATS.

### Length of hospital stay

Most (4 out of 6) of the BET studies described a significantly shorter length of hospital stay (LOS) for RATS. However, the studies by Hennon et al.,^26^ Yang et al.,^22^ and Casiraghi et al.^21^ included a comparison of RATS to VATS and open thoracotomy.

ChatGPT’s main conclusion was that there is no significant difference in the LOS after RATS or VATS. Instead, it suggested that factors such as postoperative course (air leak, pain control, complications) and patient factors (age, comorbidities, lung function) are more influential. Although ChatGPT cited 3 meta-analyses,^31^^,^^32^^,^^38^ it incorrectly referenced data from these papers.

Gemini stated that the available data on LOS is mixed, with some studies suggesting RATS offers a shorter hospital LOS^34^ and others finding no significant difference.^31^

Grok concluded that the LOS is similar between RATS and VATS, citing the RVlob trial.^13^ It also suggested that in specific scenarios, RATS may offer a shorter LOS due to lower complication rates. Grok highlighted that LOS is influenced by complications (eg, prolonged air leaks, pneumonia, atrial fibrillation), patient factors (eg, performance status, adhesions) and the surgeon/institution. It also emphasized the importance of discussing personal risk factors and the surgeon’s experience when deciding on RATS or VATS.

Copilot cited 2 studies^39^^,^^40^ suggesting that there is no significant difference in length of stay for RATS or VATS. Like Gemini and Grok, Copilot suggested RATS may offer a slightly shorter hospital stay, citing one review article^41^ but incorrectly referencing its data. The data from the review article was referenced from another article,^42^ which actually compared LOS between RATS with Open rather than RATS with VATS.

### Pain

The studies by Testori et al.^25^ and Jin et al.^28^ were the only BET papers which discussed pain. They concluded that there was no statistically significant difference in postoperative pain after RATS or VATS.

ChatGPT stated that there was no significant difference in postoperative pain between RATS and VATS. Instead, it suggested that factors such as nerve block or epidural, the number of ports/incisions and postoperative complications are more significant.

Gemini quoted an online blog^43^ suggesting there is less postoperative pain for RATS and also a study contradicting this^44^ which only focused on Uniportal VATS lobectomy. Gemini concluded that a single-port VATS procedure might offer less postoperative pain compared to RATS due to less chest wall trauma.

Grok cited the RVlob trial,^28^ correctly concluding that there is no significant difference in postoperative pain but incorrectly referencing its data. Additionally, to minimize pain, Grok advised checking whether the hospital offers multimodal analgesia and encouraged a discussion of pain tolerance and comorbidities with the patient’s thoracic surgeon.

Copilot suggested that RATS offers lower immediate postoperative pain, although the study it cited^45^ actually contradicts this. Instead, the study suggests a better pain profile for VATS compared to RATS. For chronic pain, Copilot cited a study^46^ and correctly referenced its data, suggesting a similar chronic pain profile for RATS and VATS.

## DISCUSSION

With the increasing use of low-dose computed tomography (LD-CT) for lung cancer screening,^47^ there has been a rise in the incidence of stage I NSCLC cases.^48^ In patients with Stage I NSCLC tumours > 2 cm (T1c) N0M0 (Stage 1A3), lobectomy is recommended over sublobar resection.^49^ We chose this scenario to simplify our LLM search and BET by excluding the possibility of sublobar resections^50^^,^^51^ and the option of neoadjuvant treatment.^52^ Minimally invasive techniques (RATS and VATS) are favoured over open thoracotomy.^49^^,^^53^ We wanted to evaluate which minimally invasive approach (RATS or VATS) offers better outcomes for pain, length of stay, and mortality by asking the LLMs and then comparing their outputs to a traditional BET search (LLM-BET).

According to the BET search, there are no major differences between RATS and VATS lobectomies for our chosen outcomes in early node-negative NSCLC (T1cN0M0) apart from a shorter length of hospital stay following RATS. Most of the LLMs also suggested a shorter length of hospital stay following RATS apart from ChatGPT which stated that there was no significant difference. However, there may be differences between the surgical approaches for other outcomes such as operative blood loss or lymph node yield. For instance, RATS is said to have a higher nodal yield.^32^^,^^38^

The LLM outputs were almost instantaneous. Certainly, all our primary and follow-up outputs were generated in less than a minute. In comparison, the BET component took 2 authors approximately 48 hours to complete, including cross-checking database searches manually. Additional limitations for BETs include subjectivity over which papers represent the best evidence and its time-consuming nature. Arguably, the biggest limitation for BETs is the human oversight required as opposed to the simplicity of an LLM prompt. The rapid response to LLM inputs is an obvious added advantage.

Overall, the LLMs were broadly similar in their responses. However, we noticed several issues with the LLMs in their current models (see “LLM VERSIONS USED”). As we discuss below, these might adversely affect their use in helping patients understand their disease and choose between management options.

LLMs may not provide relevant evidence. This was common to all the LLMs we used, as they chose to reference papers which didn’t solely focus on stage I NSCLC patients, early lung cancer, or even include analyses of the relevant subgroup. Given that our clinical scenario focused on a patient with stage IA3 (T1cN0M0) NSCLC, the conclusions reached by the LLMs may not be entirely accurate for our question.

Additionally, some LLMs suffer from hallucinations. These are a recognized problem with LLMs.^10–12^ LLMs are statistical machines which can predict the most probable word next in a sequence.^54^ As LLMs are based on learnt associations, they can sometimes produce answers which sound plausible but are actually incorrect.^12^ In fact, according to a preprint by OpenAI, LLMs are often rewarded for guessing a response versus acknowledging uncertainty.^55^ In some of the outputs in our scenario, particularly with ChatGPT and Grok, the LLM would include made-up data from a paper to support its claim or include links to completely irrelevant articles.^16–19^ LLM hallucinations of incorrect citations and data are dangerous as they may misinform the reader and falsely reassure them that the data is based on published evidence.

A grey area with the use of LLMs is their ability to access published and peer-reviewed literature that is restricted by a paywall or subscription. For example, ChatGPT may have limited access to some journals and articles which are copyrighted.^56^ This will limit LLMs in their ability to comprehensively review the available literature and produce an accurate response to medical questions. Furthermore, it can create a vicious cycle. By restricting the literature available, it is more likely that the LLM will reference data from sources which might not be entirely relevant to the user’s prompts. Interestingly, in a working paper published by Rosenblat et al.^57^ OpenAI LLMs were investigated to see if they were trained on copyrighted material. The GPT-4o model was shown to have a strong recognition of content behind a paywall, thus raising the question of whether LLMs would be able to access the entirety of the relevant information on a specific medical topic.

In a recent systematic review LLMs were shown to have several applications in lung cancer.^58^ For instance, LLMs were used to analyse electronic medical records allowing them to clinically stage and predict lymph node metastasis in patients with early lung cancer diagnosis.^58^ LLMs have also been assessed for their potential to support clinical decision-making in oncology and have shown mixed results.^59^^,^^60^ MEREDITH is an LLM system which is trained on data sources including PubMed-indexed clinical studies as well as trial databases to recommend treatments in precision oncology.^59^ One study compared MEREDITH with medical expert opinions for treatment advice in 10 fictional oncologic clinical scenarios.^59^ Recommendations made by MEREDITH were shown to have a high concordance (94.7%) with the medical expert opinions and there were no reported hallucinations. This study highlighted the importance of LLM training data. By providing LLMs access to peer-reviewed clinical data sources used by medical experts, as was the case with MEREDITH, healthcare-related LLMs may complement clinical practice.

However, who should be held accountable if mistakes occur in clinical practice as a result of the use of LLMs? This is why it is important to emphasize the use of LLMs simply as a tool for obtaining additional information and not the final arbiter for making clinical decisions. They should help inform practice where possible rather than dictate it, allowing both clinicians and patients to learn more about medicine and medical topics.

In future versions of LLMs, the issue of hallucinations should be addressed so that they correctly reference evidence-based literature. Additionally, LLMs should be able to access the most up-to-date peer-reviewed clinical literature so that accurate and precise conclusions can be made. There are other barriers to the implementation of LLMs including the risk of biased training data.^61^ Specific patient populations may be underrepresented resulting in skewed training data for LLMs that can lead to flawed conclusions.^62^ Therefore, it is not enough for LLMs to be allowed access to peer-reviewed literature, but the literature should also be representative to avoid bias. If these improvements are made, then we believe that LLMs may be able to supplement traditional evidence synthesis and streamline the workflow for BETs and potentially review articles. However, at the moment they are simply unreliable for summarizing evidence. Perhaps, it would be wise to inform patients about the risks of relying on LLMs to make clinical decisions and advise them to use LLMs simply as a supplementary tool. This should help them during the consultation with a clinician.

We propose a standardized LLM-BET where LLM outputs are benchmarked against a traditional BET search to investigate the capabilities of LLMs in influencing patient decisions. We hope that future BETs can be adapted to include a comparison to searches by LLMs. This would help clinicians understand the scientific perspective of patients using LLMs to inform their understanding of their disease and recommended treatments. Furthermore, LLM-BETs will highlight where there is a weakness in the use of LLMs for specific research questions. For instance, this could be due to a lack of specific literature on the topic or increased hallucinations.

This benchmarking protocol can be applied to diverse clinical scenarios and associated questions. However, the same questions must be posed to each LLM and only relevant additional outputs accepted. Each LLM should be assessed according to the criteria listed (**Table 1**) and the cited references carefully reviewed to see if the data is correctly quoted.

It is important to understand that this is our personal and contemporaneous experience of questioning LLMs. Asking the same questions again to a future iteration of either the same or a different LLM might produce a different response.^63^ We believe that at the moment, LLMs cannot replace BETs due to their potential lack of access to literature, hallucinations and their lack of sensitivity in selecting suitable research papers.

## CONCLUSION

This study proposes a framework which could be used to benchmark LLMs with a BET for specific clinical scenarios. They may also be used to analyse outputs of different models of current and future LLMs. Our BET determined that there are no significant differences between RATS and VATS for T1cN0M0 NSCLC apart from a shorter LOS for RATS. The LLMs had similar responses although there were several limitations in their use. These included hallucinations, lack of sensitivity for specific literature and a potential lack of access to evidence-based literature. For these reasons, we believe that LLMs in their current models may not be entirely reliable for answering clinical questions. LLMs are increasingly popular among patients and therefore as clinicians we cannot ignore them. However, to reduce and eliminate their inherent weaknesses we have to be vigilant and exercise an element of regulatory oversight in their development.

## LLM VERSIONS USED

We used the web-based versions of ChatGPT-5, Microsoft 365 Copilot, Grok 4 auto and Gemini 2.5 flash for our questions and their outputs. We did not select any customized modes for the LLMs.

## Supplementary Material

ivag038_Supplementary_Data

## Data Availability

The data underlying this article are available in the article and in its online supplementary material.

## References

[1] Accessed September 1, 2025. https://www.elastic.co/what-is/large-language-models#difference-between-large-language-models-and-generative-ai

[2] Chen D , Parsa R, Swanson K, et al Large language models in oncology: a review. BMJ Oncol. 2025;4:e000759. 10.1136/bmjonc-2025-000759 PMC12164365 40519217

[3] Busch F , Hoffmann L, Rueger C, et al Current applications and challenges in large language models for patient care: a systematic review. Commun Med (Lond). 2025;5:26. 10.1038/s43856-024-00717-2 39838160 PMC11751060

[4] Accessed September 1, 2025. https://www.capgemini.com/wp-content/uploads/2025/01/01_09_Capgemini-Press-release_Consumer-Trends-CRI-report-1.pdf

[5] Accessed January 4, 2026. https://www.datastudios.org/post/the-most-used-ai-chatbots-in-2025-global-usage-trends-and-platform-comparisons-of-chatgpt-gemini

[6] Accessed January 4, 2026. https://skaled.com/insights/chatpgt-vs-gemini-vs-copilot-for-sales/

[7] Accessed January 4, 2026. https://jrsdynamics.com/copilot-vs-chatgpt-vs-gemini/

[8] Accessed January 4, 2026. https://x.ai/

[9] Dunning J , Prendergast B, Mackway-Jones K. Towards evidence-based medicine in cardiothoracic surgery: best BETS. Interact CardioVasc Thorac Surg. 2003;2:405-409. 17670084 10.1016/S1569-9293(03)00191-9

[10] Huang L, Weijiang Y, Weihong Z, et al. Accessed September 23, 2025. https://arxiv.org/abs/2311.05232, preprint: not peer reviewed.

[11] Cossio M. Accessed September 23, 2025. https://arxiv.org/abs/2508.01781, preprint: not peer reviewed.

[12] Gibney E. Can researchers stop AI making up citations? Nature. 2025;645:569-570. 10.1038/d41586-025-02853-8 40921873

[13] Niu Z , Cao Y, Du M, et al Robotic-assisted versus video-assisted lobectomy for resectable non-small-cell lung cancer: the RVlob randomized controlled trial. EClinicalMedicine. 2024;74:102707. 10.1016/j.eclinm.2024.102707 39105193 PMC11299594

[14] Terra RM , Araujo PHXN, Lauricella LL, Campos JRM, Trindade JRM, Pêgo-Fernandes PM. A Brazilian randomized study: robotic-assisted vs. video-assisted lung lobectomy outcomes (BRAVO trial). J Bras Pneumol. 2022;48:e20210464. 10.36416/1806-3756/e20210464 35830078 PMC9262442

[15] Kent MS , Hartwig MG, Vallières E, et al Pulmonary open, robotic, and thoracoscopic lobectomy (PORTaL) study: an analysis of 5721 cases. Ann Surg. 2023;277:528-533. 10.1097/SLA.0000000000005115 34534988 PMC9891268

[16] Notice of correction. J Thorac Cardiovasc Surg. 2023;166:1824. 10.1016/j.jtcvs.2023.08.042 37676206

[17] Dumfarth J , Stastny L, Gasser S, Grimm M. Cardiopulmonary arrest in acute type a aortic dissection-the call for a treatment algorithm! Eur J Cardiothorac Surg. 2023;63:ezad123. 10.1093/ejcts/ezad123 37018149

[18] Hadaya J , Chervu NL, Ebrahimian S, et al Clinical outcomes and costs of robotic-assisted vs conventional mitral valve repair: a national analysis. Ann Thorac Surg. 2025;119:1011-1019. 10.1016/j.athoracsur.2024.11.005 39536852

[19] Sharma V , Ricketts HC, McCombie L, et al A total diet replacement weight management program for difficult-to-treat asthma associated with obesity: a randomized controlled feasibility trial. Chest. 2023;163:1026-1037. 10.1016/j.chest.2023.01.015 36649753 PMC10808069

[20] Zhang J , Wang Z, Wang Y, et al Comparison of long-term survival between robotic and video-assisted lobectomy for stage I NSCLC with radiologic solid tumors: a propensity score matching study. Clin Lung Cancer. 2025;26:e63-e72. 10.1016/j.cllc.2024.10.004 39516169

[21] Casiraghi M , Mariolo AV, Mohamed S, et al Long-term outcomes of robotic-assisted, video-assisted and open surgery in non-small cell lung cancer: a matched analysis. J Clin Med. 2022;11:3363. 10.3390/jcm11123363 35743434 PMC9225497

[22] Yang HX , Woo KM, Sima CS, et al Long-term survival based on the surgical approach to lobectomy for clinical stage I nonsmall cell lung cancer: comparison of robotic, video-assisted thoracic surgery, and thoracotomy lobectomy. Ann Surg. 2017;265:431-437. 10.1097/SLA.0000000000001708 28059973 PMC5033685

[23] Cui Y , Grogan EL, Deppen SA, et al Mortality for robotic- vs video-assisted lobectomy-treated stage i non-small cell lung cancer patients. JNCI Cancer Spectr. 2020;4:pkaa028. 10.1093/jncics/pkaa028 33215060 PMC7660043

[24] Li JT , Liu PY, Huang J, et al Perioperative outcomes of radical lobectomies using robotic-assisted thoracoscopic technique vs. video-assisted thoracoscopic technique: retrospective study of 1,075 consecutive p-stage I non-small cell lung cancer cases. J Thorac Dis. 2019;11:882-891. 10.21037/jtd.2019.01.78 31019777 PMC6462703

[25] Testori A , Giudici VM, Voulaz E, Alloisio M, Bottoni E. Robotic and video-assisted thoracic surgery for early-stage lung cancer: comparison of long-term pain at a single centre. J Clin Med. 2022;11:1108. 10.3390/jcm11041108 35207381 PMC8877832

[26] Hennon MW , DeGraaff LH, Groman A, Demmy TL, Yendamuri S. The association of nodal upstaging with surgical approach and its impact on long-term survival after resection of non-small-cell lung cancer. Eur J Cardiothorac Surg. 2020;57:888-895. 10.1093/ejcts/ezz320 31764992 PMC7179045

[27] Yang CF , Sun Z, Speicher PJ, et al Use and outcomes of minimally invasive lobectomy for stage I non-small cell lung cancer in the national cancer data base. Ann Thorac Surg. 2016;101:1037-1042. 10.1016/j.athoracsur.2015.11.018 26822346 PMC4763985

[28] Jin R , Zhang Z, Zheng Y, et al Health-related quality of life following robotic-assisted or video-assisted lobectomy in patients with non-small cell lung cancer: results from the RVlob randomized clinical trial. Chest. 2023;163:1576-1588. 10.1016/j.chest.2022.12.037 36621757

[29] Fabbri G , Femia F, Lampridis S, et al Long-term oncologic outcomes in robot-assisted and video-assisted lobectomies for non-small cell lung cancer. J Clin Med. 2023;12:6609. 10.3390/jcm12206609 37892747 PMC10607558

[30] Kneuertz PJ , D’Souza DM, Richardson M, Abdel-Rasoul M, Moffatt-Bruce SD, Merritt RE. Long-term oncologic outcomes after robotic lobectomy for early-stage non-small-cell lung cancer versus video-assisted thoracoscopic and open thoracotomy approach. Clin Lung Cancer. 2020;21:214-224.e2. 10.1016/j.cllc.2019.10.004 31685354

[31] Huang S , Huang X, Huang Z, Luo R, Liang W. Comparison of robot-assisted thoracic surgery versus video-assisted thoracic surgery in the treatment of lung cancer: a systematic review and meta-analysis of prospective studies. Front Oncol. 2023;13:1271709. 10.3389/fonc.2023.1271709 38023124 PMC10646752

[32] Ma J , Li X, Zhao S, Wang J, Zhang W, Sun G. Robot-assisted thoracic surgery versus video-assisted thoracic surgery for lung lobectomy or segmentectomy in patients with non-small cell lung cancer: a meta-analysis. BMC Cancer. 2021;21:498. 10.1186/s12885-021-08241-5 33941112 PMC8094485

[33] Catelli C , Corzani R, Zanfrini E, et al Robotic-assisted (RATS) versus video-assisted (VATS) lobectomy: a monocentric prospective randomized trial. Eur J Surg Oncol. 2023;49:107256. 10.1016/j.ejso.2023.107256 37925829

[34] Li C , Hu Y, Huang J, et al Comparison of robotic-assisted lobectomy with video-assisted thoracic surgery for stage IIB-IIIA non-small cell lung cancer. Transl Lung Cancer Res. 2019;8:820-828. 10.21037/tlcr.2019.10.15 32010560 PMC6976359

[35] Kent MS , Hartwig MG, Vallières E, et al Pulmonary open, robotic, and thoracoscopic lobectomy (PORTaL) study: survival analysis of 6646 cases. Ann Surg. 2023;277:1002-1009. 10.1097/SLA.0000000000005820 36762564

[36] O’Sullivan KE , Kreaden US, Hebert AE, Eaton D, Redmond KC. A systematic review and meta-analysis of robotic versus open and video-assisted thoracoscopic surgery approaches for lobectomy. Interact CardioVasc Thorac Surg. 2019;28:526-534. 10.1093/icvts/ivy315 30496420

[37] Wu H , Jin R, Yang S, Park BJ, Li H. Long-term and short-term outcomes of robot- versus video-assisted anatomic lung resection in lung cancer: a systematic review and meta-analysis. Eur J Cardiothorac Surg. 2021;59:732-740. 10.1093/ejcts/ezaa426 33367615

[38] Mao J , Tang Z, Mi Y, et al Robotic and video-assisted lobectomy/segmentectomy for non-small cell lung cancer have similar perioperative outcomes: a systematic review and meta-analysis. Transl Cancer Res. 2021;10:3883-3893. 10.21037/tcr-21-646 35116688 PMC8798077

[39] Ueno H , Imamura Y, Okado S, et al Lobectomy for primary lung cancer: a comparison of perioperative and postoperative outcomes between robot-assisted thoracic surgery and video-assisted thoracic surgery. Surg Today. 2025;55:1162-1172. 10.1007/s00595-025-03000-6 39960546 PMC12339585

[40] Otorkpa MJ , Arif S, Gooseman M, et al Comparing perioperative outcomes in video-assisted thoracic surgery and robot-assisted thoracic surgery in lung cancer surgeries: a single-Centre experience. Cardiothorac Surg. 2025;33:9. 10.1186/s43057-025-00153-5

[41] Veronesi G. Robotic lobectomy and segmentectomy for lung cancer: results and operating technique. J Thorac Dis. 2015;7:S122-S130. 10.3978/j.issn.2072-1439.2015.04.34 25984357 PMC4419035

[42] Veronesi G , Galetta D, Maisonneuve P, et al Four-arm robotic lobectomy for the treatment of early-stage lung cancer. J Thorac Cardiovasc Surg. 2010;140:19-25. 10.1016/j.jtcvs.2009.10.025 20038475

[43] Accessed September 24, 2025. https://chestsurgeryindia.com/blog/robotic-lobectomy-vs-vats-lung-cancer-delhi-ncr

[44] Tulinský L , Jarošová N, Adamica D, et al Pain and recovery after robotic vs. uniportal lobectomy for lung cancer: a comparative analysis. Surg Endosc. 2025;39:7181-7190. 10.1007/s00464-025-12083-8 40854991 PMC12618369

[45] Novellis P , Maisonneuve P, Dieci E, et al Quality of life, postoperative pain, and lymph node dissection in a robotic approach compared to VATS and OPEN for early stage lung cancer. J Clin Med. 2021;10:1687. 10.3390/jcm10081687 33920023 PMC8071041

[46] Qsous G , Downes A, Carroll B, et al A comparison of the differences in postoperative chronic pain between video-assisted and robotic-assisted approaches in thoracic surgery. Cureus. 2022;14:e31688. 10.7759/cureus.31688 36561601 PMC9764266

[47] Verma N , Zanon M, Torri G, Hochhegger B. Low-dose CT for lung cancer screening: updates and clinical impact. Semin Roentgenol. 2025;60:357-364. 10.1053/j.ro.2025.05.002 41115735

[48] Singareddy A , Flanagan ME, Samson PP, et al Trends in stage I lung cancer. Clin Lung Cancer. 2023;24:114-119. 10.1016/j.cllc.2022.11.005 36504141

[49] Howington J , Souter LH, Arenberg D, et al Management of patients with early-stage non-small cell lung cancer: an American College of Chest Physicians Clinical Practice guideline. Chest. 2025;168:810-827. 10.1016/j.chest.2025.06.023 40562304

[50] Altorki N , Wang X, Kozono D, et al Lobar or sublobar resection for peripheral stage IA non-small-cell lung cancer. N Engl J Med. 2023;388:489-498. 10.1056/NEJMoa2212083 36780674 PMC10036605

[51] Saji H , Okada M, Tsuboi M, , et al; West Japan Oncology Group and Japan Clinical Oncology Group. Segmentectomy versus lobectomy in small-sized peripheral non-small-cell lung cancer (JCOG0802/WJOG4607L): a multicentre, open-label, phase 3, randomised, controlled, non-inferiority trial. Lancet. 2022;399:1607-1617. 10.1016/S0140-6736(21)02333-3 35461558

[52] Forde PM , Spicer J, Lu S, et al; CheckMate 816 Investigators. Neoadjuvant nivolumab plus chemotherapy in resectable lung cancer. N Engl J Med. 2022;386:1973-1985. 10.1056/NEJMoa2202170 35403841 PMC9844511

[53] Chaudhuri N , Chaudhuri V. Surgical management of early lung cancer: is robotic-assisted thoracic surgery or video-assisted thoracoscopic surgery better? Chest. 2026;169 2:e86-e87. 10.1016/j.chest.2025.09.135 41672672

[54] Stryker C. Accessed September 27, 2025. https://www.ibm.com/think/topics/large-language-models

[55] Accessed September 27, 2025. https://openai.com/index/why-language-models-hallucinate/

[56] Biswas SS. ChatGPT for research and publication: a step-by-step guide. J Pediatr Pharmacol Ther. 2023;28:576-584. 10.5863/1551-6776-28.6.576 38130350 PMC10731938

[57] Sruly R , Tim O, Strauss I. “Beyond public access in LLM pre-training data: non-public book content in OpenAI’s models.” SSRC AI Disclosures Project Working Paper Series (SSRC AI WP 2025-04), Social Science Research Council, April 2025. Accessed September 27, 2025. https://www.ssrc.org/publications/beyond-public-access-in-llm-pre-training-data-non-public-bookcontent-in-openais-models/

[58] Zhong R , Chen S, Li Z, et al Large language models in lung cancer: systematic review. J Med Internet Res. 2025;27:e74177. 10.2196/74177 41026980 PMC12483341

[59] Lammert J , Dreyer T, Mathes S, et al Expert-guided large language models for clinical decision support in precision oncology. JCO Precis Oncol. 2024;8:e2400478. 10.1200/PO-24-00478 39475661

[60] Benary M , Wang XD, Schmidt M, et al Leveraging large language models for decision support in personalized oncology. JAMA Netw Open. 2023;6:e2343689. 10.1001/jamanetworkopen.2023.43689 37976064 PMC10656647

[61] Luo Y , Hooshangnejad H, Ngwa W, Ding K. Opportunities and challenges in lung cancer care in the era of large language models and vision language models. Transl Lung Cancer Res. 2025;14:1830-1847. 10.21037/tlcr-24-801 40535072 PMC12170128

[62] Mahajan A , Obermeyer Z, Daneshjou R, Lester J, Powell D. Cognitive bias in clinical large language models. NPJ Digit Med. 2025;8:428. 10.1038/s41746-025-01790-0 40640549 PMC12246145

[63] Wang L , Chen X, Deng X, et al Prompt engineering in consistency and reliability with the evidence-based guideline for LLMs. NPJ Digit Med. 2024;7:41. 10.1038/s41746-024-01029-4 38378899 PMC10879172

