# Supplementary material for: Benchmarking Large Language Models Using a Best Evidence Topic Report in a Patient With Early Non-Small Cell Lung Cancer
Source: Interdiscip Cardiovasc Thorac Surg. 2026 Feb 6;41(2):ivag038. doi: 10.1093/icvts/ivag038 (PMC12927417; doi:10.1093/icvts/ivag038)
Supplement: ivag038_Supplementary_Data [file ivag038_supplementary_data.zip › Appendix 2 LLM BET .docx]

**APPENDIX 2 – References cited by LLM Outputs**

**ChatGPT:**

1. Huang S, Huang X, Huang Z, Luo R, Liang W. Comparison of robot-assisted thoracic surgery versus video-assisted thoracic surgery in the treatment of lung cancer: a systematic review and meta-analysis of prospective studies. *Front Oncol*. 2023;13:1271709. Published 2023 Oct 30. doi:10.3389/fonc.2023.1271709
2. <https://en.wikipedia.org/wiki/VATS_lobectomy> (accessed 23 Sep 2025)
3. Ma J, Li X, Zhao S, Wang J, Zhang W, Sun G. Robot-assisted thoracic surgery versus video-assisted thoracic surgery for lung lobectomy or segmentectomy in patients with non-small cell lung cancer: a meta-analysis. *BMC Cancer*. 2021;21(1):498. Published 2021 May 3. doi:10.1186/s12885-021-08241-5
4. Mao J, Tang Z, Mi Y, et al. Robotic and video-assisted lobectomy/segmentectomy for non-small cell lung cancer have similar perioperative outcomes: a systematic review and meta-analysis. Transl Cancer Res. 2021;10(9):3883-3893. doi:10.21037/tcr-21-646
5. <https://en.wikipedia.org/wiki/Lobectomy> (accessed 23 Sep 2025)
6. Tasoudis PT, Diehl JN, Merlo A, Long JM. Long-term outcomes of robotic versus video-assisted pulmonary lobectomy for non-small cell lung cancer: systematic review and meta-analysis of reconstructed patient data. J Thorac Dis. 2023;15(10):5700-5713. doi:10.21037/jtd-23-582
7. Karuppannan M, Taylor M. Examining long-term outcomes after robotic versus video-assisted lung resection. Video-assist Thorac Surg 2025;10:9.
8. Casiraghi M, Mariolo AV, Mohamed S, Sedda G, Maisonneuve P, Mazzella A, Lo Iacono G, Petrella F, Spaggiari L. Long-Term Outcomes of Robotic-Assisted, Video-Assisted and Open Surgery in Non-Small Cell Lung Cancer: A Matched Analysis. Journal of Clinical Medicine. 2022; 11(12):3363. <https://doi.org/10.3390/jcm11123363>
9. Cui Y, Grogan EL, Deppen SA, et al. Mortality for Robotic- vs Video-Assisted Lobectomy-Treated Stage I Non-Small Cell Lung Cancer Patients. JNCI Cancer Spectr. 2020;4(5):pkaa028. Published 2020 Apr 15. doi:10.1093/jncics/pkaa028
10. Sesti J, Langan RC, Bell J, et al. A Comparative Analysis of Long-Term Survival of Robotic Versus Thoracoscopic Lobectomy. Ann Thorac Surg. 2020;110(4):1139-1146. doi:10.1016/j.athoracsur.2020.03.085
11. O'Sullivan KE, Kreaden US, Hebert AE, Eaton D, Redmond KC. A systematic review and meta-analysis of robotic versus open and video-assisted thoracoscopic surgery approaches for lobectomy. Interact Cardiovasc Thorac Surg. 2019;28(4):526-534. doi:10.1093/icvts/ivy315
12. Montagne F, Bottet B, Sarsam M, Mbadinga F, Chaari Z, Rinieri P, Melki J, Peillon C, Baste JM. Robotic versus open and video-assisted thoracoscopic surgery approaches for lobectomy. Mini-invasive Surg. 2020;4:17. <http://dx.doi.org/10.20517/2574-1225.2019.74>

**Gemini:**

1. Wang Y, Meng C, Shi L, Gu S, Fan X, Wang Q. Short-term outcomes of robotic- vs. television-assisted thoracoscopic segmental lung resection for early-stage non-small-cell lung cancer in the day surgery models. J Thorac Dis. 2024;16(11):7257-7270. doi:10.21037/jtd-24-1020
2. Zheng L, Song P, Jiang Y, et al. Outcomes and quality of life after Robot-assisted lobectomy/segmentectomy for lung cancer compared to video-assisted thoracoscopic surgery: both three-port procedures performed by a single surgeon. J Thorac Dis. 2022;14(3):689-698. doi:10.21037/jtd-22-238
3. Muriana P, Veronesi G. Oncological clearance of minimally invasive lobectomy for clinical N0 non-small cell lung cancer: the role of robotic surgery. Video-assist Thorac Surg 2021;6:15. doi: 10.21037/vats-20-42
4. https://chestsurgeryindia.com/blog/robotic-lobectomy-vs-vats-lung-cancer-delhi-ncr (accessed 23 Sep 2025)
5. Gallina FT, Tajè R, Forcella D, et al. Perioperative outcomes of robotic lobectomy for early-stage non-small cell lung cancer in elderly patients. *Front Oncol*. 2022;12:1055418. Published 2022 Nov 28. doi:10.3389/fonc.2022.1055418
6. Gallina FT, Tajè R, Forcella D, et al. Oncological Outcomes of Robotic Lobectomy and Radical Lymphadenectomy for Early-Stage Non-Small Cell Lung Cancer. J Clin Med. 2022;11(8):2173. Published 2022 Apr 13. doi:10.3390/jcm11082173
7. <https://go2.org/treatments-and-side-effects/treatment-options/surgery/#:~:text=Minimally%20invasive%20surgery,may%20not%20be%20available%20everywhere>. (accessed 23 Sep 2025)
8. Li C, Hu Y, Huang J, et al. Comparison of robotic-assisted lobectomy with video-assisted thoracic surgery for stage IIB-IIIA non-small cell lung cancer. *Transl Lung Cancer Res*. 2019;8(6):820-828. doi:10.21037/tlcr.2019.10.15
9. <https://my.clevelandclinic.org/health/treatments/17608-lobectomy> (accessed 23 Sep 2025)
10. Lampridis S, Maraschi A, Le Reun C, Routledge T, Billè A. Robotic versus Video-Assisted Thoracic Surgery for Lung Cancer: Short-Term Outcomes of a Propensity Matched Analysis. Cancers (Basel). 2023;15(8):2391. Published 2023 Apr 21. doi:10.3390/cancers15082391
11. <https://my.clevelandclinic.org/health/treatments/17617-video-assisted-thoracic-surgery-vats> (accessed 23 Sep 2025)
12. Huang S, Huang X, Huang Z, Luo R, Liang W. Comparison of robot-assisted thoracic surgery versus video-assisted thoracic surgery in the treatment of lung cancer: a systematic review and meta-analysis of prospective studies. *Front Oncol*. 2023;13:1271709. Published 2023 Oct 30. doi:10.3389/fonc.2023.1271709
13. Augustin F, Bodner J, Maier H, et al. Robotic-assisted minimally invasive vs. thoracoscopic lung lobectomy: comparison of perioperative results in a learning curve setting. *Langenbecks Arch Surg*. 2013;398(6):895-901. doi:10.1007/s00423-013-1090-5
14. García-Pérez A, Delgado Roel M, Minasyan A, Durán HMT, Fernández-Prado R, Fieira Costa EM, Fernández Vago L, Sánchez Valenzuela IA, Gamborino MC, de la Torre Bravos M. Robotic-assisted thoracic surgery (RATS) versus video-assisted thoracic surgery (VATS) in oncological lung resections—comparative study during initial experience of 3 surgeons. Video-assist Thorac Surg 2025;10:3. doi: 10.21037/vats-24-13
15. <https://chestsurgeryindia.com/> (accessed 23 Sep 2025)
16. O'Sullivan KE, Kreaden US, Hebert AE, Eaton D, Redmond KC. A systematic review and meta-analysis of robotic versus open and video-assisted thoracoscopic surgery approaches for lobectomy. *Interact Cardiovasc Thorac Surg*. 2019;28(4):526-534. doi:10.1093/icvts/ivy315
17. Montagne F, Bottet B, Sarsam M, Mbadinga F, Chaari Z, Rinieri P, Melki J, Peillon C, Baste JM. Robotic versus open and video-assisted thoracoscopic surgery approaches for lobectomy. Mini-invasive Surg. 2020;4:17. <http://dx.doi.org/10.20517/2574-1225.2019.74>
18. Karuppannan M, Taylor M. Examining long-term outcomes after robotic versus video-assisted lung resection. Video-assist Thorac Surg 2025;10:9.
19. Shindo Y, Miyajima M, Nakamura Y, et al. Number of lymph nodes dissected and upstaging rate of the N factor in robot-assisted thoracic surgery versus video-assisted thoracic surgery for patients with cN0 primary lung cancer. *Surg Today*. 2023;53(4):428-434. doi:10.1007/s00595-022-02578-5
20. Tasoudis PT, Diehl JN, Merlo A, Long JM. Long-term outcomes of robotic versus video-assisted pulmonary lobectomy for non-small cell lung cancer: systematic review and meta-analysis of reconstructed patient data. J Thorac Dis. 2023;15(10):5700-5713. doi:10.21037/jtd-23-582
21. Wong LY, Oh DS. Scoping review on long term oncologic outcomes in robotic-assisted lobectomy. Video-assist Thorac Surg 2023;8:28. doi: 10.21037/vats-23-26
22. Tulinský L, Jarošová N, Adamica D, et al. Pain and recovery after robotic vs. uniportal lobectomy for lung cancer: a comparative analysis. *Surg Endosc*. Published online August 25, 2025. doi:10.1007/s00464-025-12083-8
23. Tokuishi K, Wakahara JI, Ueda Y, et al. Comparison of postoperative pain between robotic and uniportal video-assisted thoracic surgery for anatomic lung resection in patients with stage I lung cancer. *Gen Thorac Cardiovasc Surg*. 2025;73(9):692-699. doi:10.1007/s11748-025-02129-y
24. Catelli C, Corzani R, Zanfrini E, et al. RoboticAssisted (RATS) versus Video-Assisted (VATS) lobectomy: A monocentric prospective randomized trial. Eur J Surg Oncol. 2023;49(12):107256. doi:10.1016/j.ejso.2023.107256
25. <https://chestsurgeryindia.com/blog/robotic-lobectomy-vs-vats-lung-cancer-delhi-ncr> (accessed 24 Sep 2025)

**Grok:**

1. Notice of Correction. *J Thorac Cardiovasc Surg*. 2023;166(6):1824. doi:10.1016/j.jtcvs.2023.08.042 (accessed 23 Sep 2025)
2. Dumfarth J, Stastny L, Gasser S, Grimm M. Cardiopulmonary arrest in acute type A aortic dissection-the call for a treatment algorithm!. *Eur J Cardiothorac Surg*. 2023;63(4):ezad123. doi:10.1093/ejcts/ezad123 (accessed 23 Sep 2025)
3. Hadaya J, Chervu NL, Ebrahimian S, et al. Clinical Outcomes and Costs of Robotic-assisted vs Conventional Mitral Valve Repair: A National Analysis. *Ann Thorac Surg*. 2025;119(5):1011-1019. doi:10.1016/j.athoracsur.2024.11.005 (accessed 23 Sep 2025)
4. Sharma V, Ricketts HC, McCombie L, et al. A Total Diet Replacement Weight Management Program for Difficult-to-Treat Asthma Associated With Obesity: A Randomized Controlled Feasibility Trial. *Chest*. 2023;163(5):1026-1037. doi:10.1016/j.chest.2023.01.015 (accessed 23 Sep 2025)
5. Tasoudis PT, Diehl JN, Merlo A, Long JM. Long-term outcomes of robotic versus video-assisted pulmonary lobectomy for non-small cell lung cancer: systematic review and meta-analysis of reconstructed patient data. J Thorac Dis. 2023;15(10):5700-5713. doi:10.21037/jtd-23-582
6. Niu Z, Cao Y, Du M, et al. Robotic-assisted versus video-assisted lobectomy for resectable non-small-cell lung cancer: the RVlob randomized controlled trial. *EClinicalMedicine*. 2024;74:102707. Published 2024 Jul 12. doi:10.1016/j.eclinm.2024.102707
7. Asemota N, Maraschi A, Lampridis S, et al. Comparison of Quality of Life after Robotic, Video-Assisted, and Open Surgery for Lung Cancer. *J Clin Med*. 2023;12(19):6230. Published 2023 Sep 27. doi:10.3390/jcm12196230
8. Huang L, Liang S, He J, et al. Impact of robotic-assisted versus video-assisted thoracoscopic surgery on efficacy and quality of life in patients with pulmonary opacities: a propensity score-matched analysis. *Front Oncol*. 2025;15:1598990. Published 2025 Jul 25. doi:10.3389/fonc.2025.1598990
9. Jin R, Zhang Z, Zheng Y, et al. Health-Related Quality of Life Following Robotic-Assisted or Video-Assisted Lobectomy in Patients With Non-Small Cell Lung Cancer: Results From the RVlob Randomized Clinical Trial. Chest. 2023;163(6):1576-1588. doi:10.1016/j.chest.2022.12.037

**Copilot**

1. Ma J, Li X, Zhao S, Wang J, Zhang W, Sun G. Robot-assisted thoracic surgery versus video-assisted thoracic surgery for lung lobectomy or segmentectomy in patients with non-small cell lung cancer: a meta-analysis. *BMC Cancer*. 2021;21(1):498. Published 2021 May 3. doi:10.1186/s12885-021-08241-5
2. Fabbri G, Femia F, Lampridis S, et al. Long-Term Oncologic Outcomes in Robot-Assisted and Video-Assisted Lobectomies for Non-Small Cell Lung Cancer. *J Clin Med*. 2023;12(20):6609. Published 2023 Oct 19. doi:10.3390/jcm12206609
3. Tupper HI, Lawson BL, Kipnis P, et al. Video-Assisted vs Robotic-Assisted Lung Lobectomies for Operating Room Resource Utilization and Patient Outcomes. *JAMA Netw Open*. 2024;7(5):e248881. Published 2024 May 1. doi:10.1001/jamanetworkopen.2024.8881
4. Ueno H, Imamura Y, Okado S, et al. Lobectomy for primary lung cancer: a comparison of perioperative and postoperative outcomes between robot-assisted thoracic surgery and video-assisted thoracic surgery. *Surg Today*. 2025;55(8):1162-1172. doi:10.1007/s00595-025-03000-6
5. Otorkpa, M.J., Arif, S., Gooseman, M. *et al.* Comparing perioperative outcomes in video-assisted thoracic surgery and robot-assisted thoracic surgery in lung cancer surgeries: a single-centre experience. *Cardiothorac Surg* 33, 9 (2025). <https://doi.org/10.1186/s43057-025-00153-5>
6. Veronesi G. Robotic lobectomy and segmentectomy for lung cancer: results and operating technique. *J Thorac Dis*. 2015;7(Suppl 2):S122-S130. doi:10.3978/j.issn.2072-1439.2015.04.34
7. Cui Y, Grogan EL, Deppen SA, et al. Mortality for Robotic- vs Video-Assisted Lobectomy-Treated Stage I Non-Small Cell Lung Cancer Patients. *JNCI Cancer Spectr*. 2020;4(5):pkaa028. Published 2020 Apr 15. doi:10.1093/jncics/pkaa028
8. Pan H, Zhang J, Tian Y, et al. Short- and long-term outcomes of robotic-assisted versus video-assisted thoracoscopic lobectomy in non-small cell lung cancer patients aged 35 years or younger: a real-world study with propensity score-matched analysis. *J Cancer Res Clin Oncol*. 2023;149(12):9947-9958. doi:10.1007/s00432-023-04933-6
9. Wu H, Jin R, Yang S, Park BJ, Li H. Long-term and short-term outcomes of robot- versus video-assisted anatomic lung resection in lung cancer: a systematic review and meta-analysis. *Eur J Cardiothorac Surg*. 2021;59(4):732-740. doi:10.1093/ejcts/ezaa426
10. Syrigos N, Fyta E, Goumas G, et al. Robotic-Assisted Thoracoscopic Surgery Versus Video-Assisted Thoracoscopic Surgery: Which Is the Preferred Approach for Early-Stage NSCLC?. *J Clin Med*. 2025;14(9):3032. Published 2025 Apr 28. doi:10.3390/jcm14093032
11. Novellis P, Maisonneuve P, Dieci E, et al. Quality of Life, Postoperative Pain, and Lymph Node Dissection in a Robotic Approach Compared to VATS and OPEN for Early Stage Lung Cancer. *J Clin Med*. 2021;10(8):1687. Published 2021 Apr 14. doi:10.3390/jcm10081687
12. Qsous G, Downes A, Carroll B, et al. A Comparison of the Differences in Postoperative Chronic Pain Between Video-Assisted and Robotic-Assisted Approaches in Thoracic Surgery. *Cureus*. 2022;14(11):e31688. Published 2022 Nov 20. doi:10.7759/cureus.31688
13. <https://careyourlungs.com/managing-recovery-after-lobectomy-what-to-expect-and-how-to-manage-pain/> (accessed 23 Sep 2025)
14. Tulinský L, Jarošová N, Adamica D, et al. Pain and recovery after robotic vs. uniportal lobectomy for lung cancer: a comparative analysis. *Surg Endosc*. Published online August 25, 2025. doi:10.1007/s00464-025-12083-8
15. Zhang L, Luo J, Wu X. Impact of single-port versus three-port video-assisted thoracic surgery lobectomy on postoperative pain and quality of life in early-stage non-small cell lung cancer patients: a meta-analysis. *BMC Cancer*. 2025;25(1):400. Published 2025 Mar 5. doi:10.1186/s12885-025-13755-3
