# Supplementary material for: Benchmarking Large Language Models Using a Best Evidence Topic Report in a Patient With Early Non-Small Cell Lung Cancer
Source: Interdiscip Cardiovasc Thorac Surg. 2026 Feb 6;41(2):ivag038. doi: 10.1093/icvts/ivag038 (PMC12927417; doi:10.1093/icvts/ivag038)
Supplement: ivag038_Supplementary_Data [file ivag038_supplementary_data.zip › Appendix 3 LLM BET .docx]

**APPENDIX 3**

**Checklist: Questions for Your Surgeon (Copilot)**

**Surgical Approach & Experience**

· Which approach do you recommend for my case - robotic or VATS - and why?

· How many RATS and VATS lobectomies have you performed?

· What are your complication and conversion rates for each technique?

· Is my tumor·s location or size better suited to one approach over the other?

**Hospital Capabilities**

· Does this hospital have the necessary robotic equipment and trained staff for RATS?

· Are there differences in operating room time or scheduling between RATS and VATS here?

· Is there a significant cost difference between the two approaches at this hospital?

**Recovery & Pain Management**

· What kind of pain management plan will I have after surgery?

· Do patients typically experience more pain with one approach over the other?

· How long is the expected hospital stay for each approach?

· Will I need chest tubes, and for how long with each method?

**Outcomes**

· Are there differences in lymph node removal or cancer staging accuracy between RATS and VATS?

· What are the survival and recurrence rates for each approach in patients like me?

· What is the risk of in-hospital or 30-day mortality for each technique in your experience?

**Personalized Considerations**

· Given my health profile (e.g., age, BMI, comorbidities), is one approach safer or more effective?

· Will my recovery be affected by my lifestyle or any pre-existing conditions?

· How soon can I return to normal activities after each type of surgery?
